# Exploring the Therapeutic Efficacy of Parsley (*Petroselinum crispum* Mill.) as a Functional Food: Implications in Immunological Tolerability, Reduction of Muscle Cramps, and Treatment of Dermatitis

**DOI:** 10.3390/molecules29030608

**Published:** 2024-01-27

**Authors:** Mariana Ganea, Laura Grațiela Vicaș, Octavia Gligor, Ioan Sarac, Emilian Onisan, Csaba Nagy, Corina Moisa, Timea Claudia Ghitea

**Affiliations:** 1 Department of Pharmacy, Faculty of Medicine and Pharmacy, University of Oradea, 1st Decembrie Street, 410073 Oradea, Romania; mganea@uoradea.ro (M.G.); corinamoisa@hotmail.com (C.M.); timea.ghitea@csud.uoradea.ro (T.C.G.); 2 Faculty of Pharmacy, “Iuliu Hatieganu” University of Medicine and Pharmacy, 8 Victor Babes Street, 400012 Cluj-Napoca, Romania; gligor.octavia@umfcluj.ro; 3 Department of Genetic Engineering, University of Life Sciences “King Mihai I Timișoara”, 300645 Timișoara, Romania; ioansarac@usvt.ro (I.S.); emilianonisan.sunflwoer@gmail.com (E.O.); 4 Independent Researcher, 417595 Tinca, Romania; nagycsaba95@yahoo.com

**Keywords:** *Petroselinum crispum* Mill., parsley, IgG reactions, dermatitis, muscle cramps

## Abstract

The status of parsley as a well-known folk medicine noted for its nutritional and medicinal properties prompted the exploration of its potential as a functional food and natural remedy. The paper aims to investigate the potential of parsley to enhance muscle function and alleviate psoriasiform dermatitis, eventually establishing it as a natural, well-tolerated alternative with specific benefits for both muscles and skin. This study examines the tolerability of parsley in a cohort of 937 participants by assessing immunoglobulin G (IgG) reactions. The findings reveal high tolerability, as 96.26% of participants experienced no adverse effects. Among the 902 individuals lacking hypersensitivity, 37.02% reported muscle cramps, with a notable 15.02% reduction observed in the subgroup consuming parsley juice. In the subset of 32 subjects with dermatitis, the application of parsley extract ointment led to a significant decrease in dermatological parameters (redness, thickness, scaling). While the control group exhibited improvements, statistical significance was not observed. Notably, four categories of affected area reduction were identified, with scaling demonstrating the most pronounced impact. The results propose that parsley holds promise for favorable tolerability, contributing to the alleviation of muscle cramps and presenting an effective alternative in dermatitis treatment. Nonetheless, sustained validation through long-term studies is imperative to substantiate these preliminary findings.

## 1. Introduction

Parsley (*Petroselinum crispum* Mill.) is highly appreciated not only from a gastronomical standpoint but also as a functional food with beneficial health effects. The leaves of parsley, rich in effective antioxidants, are extensively utilized in various food applications. A detailed phytochemical analysis conducted on parsley leaves from Benin revealed the presence of diverse chemical groups, including catechin tannins, gallic tannins, flavonoids, saponosides, mucilages, coumarins, reducing agents, and O-heterosides. These findings substantiate the antioxidant properties of parsley, rendering it a suitable candidate for long-term incorporation into the diet [1].

Concerning the plant’s protective effects, an ethanolic extract of parsley demonstrated a notable ability to alleviate liver toxicity, with the severity of these changes correlating with the administered dose. Furthermore, parsley extract has proven effective in preventing common side effects such as proteinuria and low hemoglobin induced by substances like paracetamol [2]. This suggests a potential role for parsley in managing liver and kidney diseases, particularly in addressing proteinuria and averting paracetamol-induced renal, hepatic, and hematologic toxicity [2].

The therapeutic efficacy of parsley extends beyond internal use. In cases of melanomas, a parsley solution exhibited systemic and local therapeutic effects comparable to conventional allopathic treatments, presenting itself as a viable alternative [3].

Historically, parsley has been recognized as a rich source of apigenin, a flavone with chemopreventive properties. Additionally, it contains elevated levels of quercetin, a flavonoid known for reducing the risk of various disorders such as neurodegenerative conditions, cancers, cardiovascular diseases, allergic disorders, thrombosis, atherosclerosis, hypertension, and arrhythmia, owing to its potent antioxidant and anti-inflammatory properties [4].

Parsley seeds, on the other hand, contributed to the development of a herbicidal product with a notable phytotoxicity, demonstrating the versatility of the plant [5].

In addition, a parsley tincture containing polyphenols, flavonoids, apioles, elemicin, and myristicin is used as a feed additive. Regulatory bodies deemed it safe for specified maximum use levels in feed for various animal species. While guidelines advise that handling should minimize exposure to apiole, elemicin, and myristicin, as parsley still poses skin and eye irritant properties, its use as a flavoring in animal feed is considered environmentally benign [6].

In conclusion, parsley emerges as a plant with exceptional food properties and high tolerability, offering therapeutic capacities in its leaves, seeds, and roots. The diverse array of substances it encompasses, subject to extraction methods, positions parsley as a versatile, functional food with potential applications across a broad spectrum of health issues. The cumulative evidence underscores the role of parsley not only as a culinary herb but also as a valuable asset in promoting health and well-being. The purpose of the paper is to identify which substance in parsley could improve targeted muscle function after internal use and improve the appearance of psoriasiform dermatitis, thus establishing parsley as a natural alternative with high tolerance and specific potential benefits for muscle function and skin health.

## 2. Results

### 2.1. IgG Test: Parsley Tolerability Assessment

**Figure 1:**
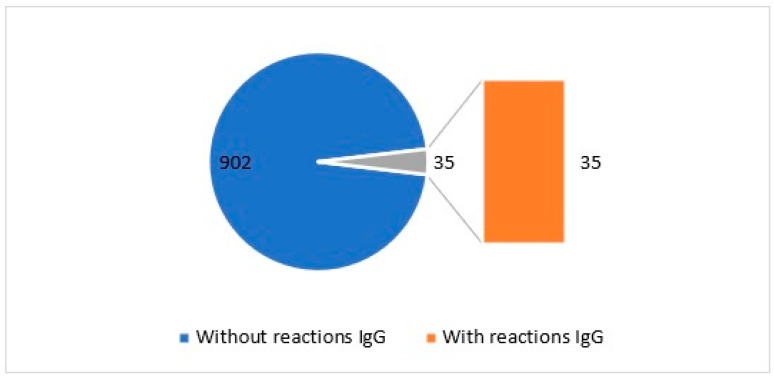
Graphical representation of individuals exhibiting IgG reactions to parsley.

The evaluation of parsley tolerability represents a crucial step in discerning locally well-tolerated products. In this comprehensive analysis, immunoglobulin G (IgG) reactions were analyzed in a cohort of 937 participants. The results revealed that 96.26% of individuals exhibited no adverse reactions following parsley consumption, as illustrated in Figure 1. Conversely, 35 individuals (3.73%) manifested reactions associated with the ingestion of this food. These findings bear significant implications for determining the tolerability threshold of parsley within the local population and may exert influence on decisions pertaining to the incorporation of this product into individual dietary regimens and overall health considerations.

### 2.2. Mineral Water for Muscle Cramps

**Figure 2:**
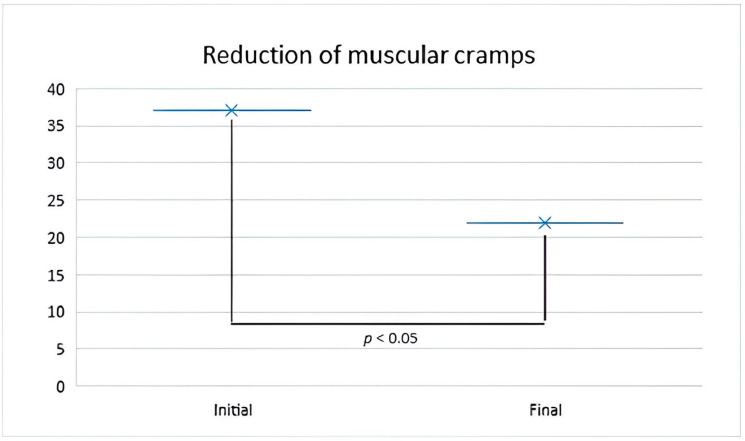
The graphic representation of the evolution of muscle cramp percentage following the consumption of a 20 mL solution of cold-pressed parsley root juice for 7 days.

Parsley, rich in minerals, is well-known for its beneficial effects in terms of remineralization. Consequently, the evolution of muscle cramp incidence was monitored based on the consumption of cold-pressed parsley root juice. Among the total of 902 participants included in the study who lacked immune reactions, 37.02% (334 people) reported experiencing muscle cramps. In contrast, only 21.95% (206 people) encountered this issue. The difference between these two proportions is statistically significant. A notable reduction in the incidence of muscle cramps by 15.02% was observed. This statistically discernible difference was assessed through Chi-square analysis, with X2 = 3212.02 and *p* = 0.006 (Figure 2).

### 2.3. Dermatological Applications

Among the 902 individuals without hypersensitivity to parsley, 32 people presented dermatological issues such as dermatitis (erythema, redness, dryness).

Out of the 32 individuals, the bioadhesive product with urea (PBU) was applied to 16, while the ointment base without any active substance was applied to the remaining 16 (control group). Ameliorations in the three parameters (redness, thickness, scaling) were observed in the 16 individuals from the research group with scaling being the parameter that demonstrated the most substantial reduction. In the control group, improvements were noted in one case. Contrariwise, the applied ointment base enhanced the appearance of dermatitis and failed to reach the threshold of statistical significance. Thus, the study included four categories: the first with “0%”; the second with “<10%”; the third category “10–29%”; the fourth “30–49%” of the affected area (Table 1).

**Figure 3:**
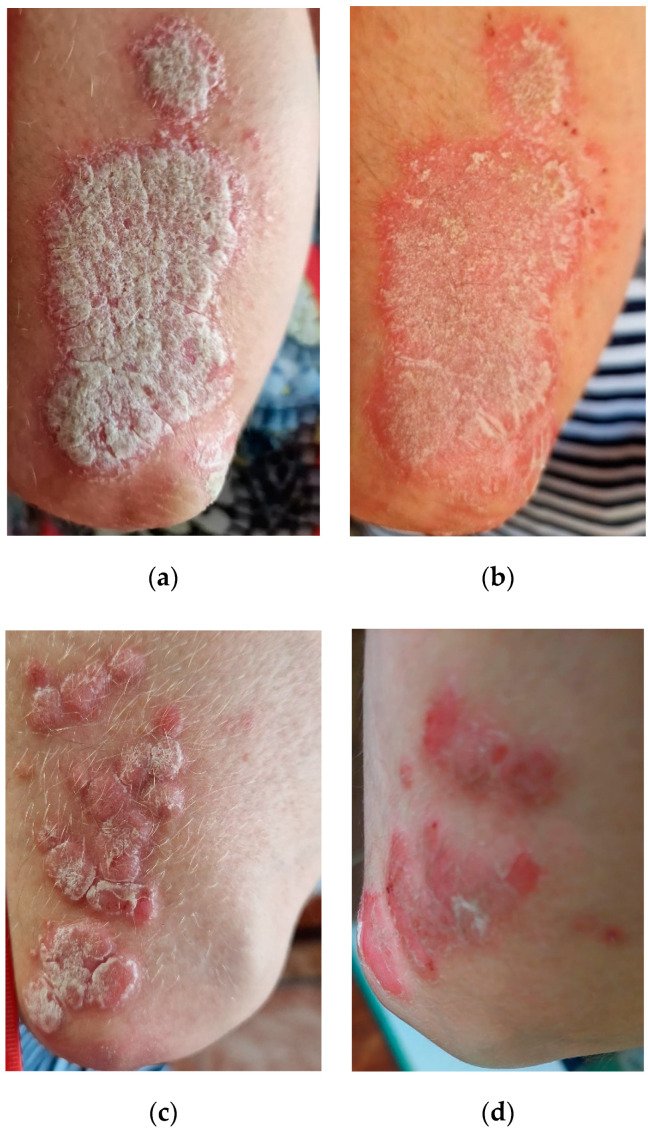
The evolution of dermatitis following the application of parsley ointment (initial (**a**), final (**b**)), prospective application of parsley and urea (initial (**c**), and final (**d**)).

Figure 3 depicts the progression of dermatitis following the application of parsley ointment with the initial state denoted as (a) and the final state as (b). Additionally, the prospective application of parsley and urea is illustrated with the initial state labeled as (c) and the final state as (d).

## 3. Discussion

**Figure 4:**
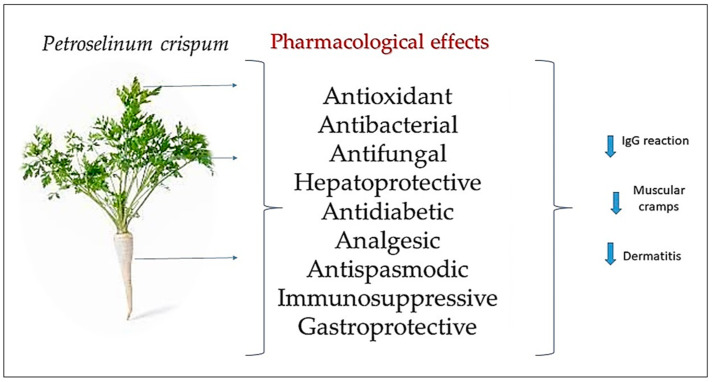
Presentation of *P. crispum* Mill. pharmacological activities.

The current study aimed to provide a detailed insight into the impact of parsley use in various pharmaceutical products. The remarkably good tolerability of parsley, observed in almost 96.26% of the participants, opens new avenues for integrating this plant into dietary regimes without inducing significant immunological reactions. In 2017, Choi et al. [7] concluded that parsley extract exhibited potent antioxidant effects and emphasized its value as a variety of functional cosmetic materials with anti-inflammatory, whitening, and wrinkle-improvement effects (Figure 4). Other studies noted that parsley essential oil may be capable of suppressing the cellular and humoral immune response [8]. This may provide an explanation for the low number of cases of IgG allergies encountered in the present study.

Twenty-nine flavonoid glycosides were identified in the aqueous extract of the leaves of *P. crispum* Mill., with apiin as the main compound isolated with a purity of 90% upon hydrolysis. Parsley extract showed a significant content of phenolic compounds, namely 12.49 ± 1.70 mg GAE/g, using the Folin–Ciocâlteu method. A high level of total flavonoids, 15.05 ± 2.20 mg QE/g, respectively, was observed. The aluminum chloride method was used in this case. Additionally, a high antioxidant activity was found in *P. crispum* Mill. extract [9,10].

The results with regard to the reduction of the incidence of muscle cramps following the consumption of parsley juice were particularly promising. This finding suggests a potential role for parsley in managing muscle symptoms, providing a natural and affordable alternative for this common problem. In 2019, data showed that parsley extract exerted hypotensive effects through vasodilating properties in an endothelium-independent pathway, as presented by Ajebli et al. [11].

Numerous studies explored the effects of parsley in various cosmetic products [12,13,14]. The topical use of *P. crispum* Mill. versus hydroquinone cream was effective in reducing epidermal melasma, as demonstrated in a randomized clinical trial [12]. Regarding the use of ointment in dermatological problems, our observations show significant potential in the relief of redness, thickness, and scaling of the skin. However, it is important to emphasize the need for further research to strengthen these findings and provide detailed information on the long-term efficacy of this treatment.

The antioxidant and, consequently, oxidative stress-reducing effects [15] were studied from the perspective of the two main components of parsley oil, apiol and myristicin [16]. Antioxidant results were also proven for parsley juice in studies published in 2016 by Papuc et al. [17] and Leahu et al. [18]. Furthermore, the roots of *P. crispum* Mill. were used as a strong diuretic, and the seeds were used as antimicrobial, antiseptic, and antispasmodic agents and in the treatment of gastrointestinal disorders, inflammation, halitosis, kidney stones, and amenorrhea. The leaves of *P. crispum* Mill. were used in the treatment of hemorrhoids and gastrointestinal disorders, as a diuretic, and as a food flavoring agent, in addition to their usual use as a culinary herb. *P. crispum* Mill. was found to possess many pharmacological effects, including antioxidant, antibacterial, antifungal, hepatoprotective, antidiabetic, analgesic, antispasmodic, immunosuppressive, and gastroprotective properties [19,20,21,22,23,24,25,26,27,28]. The results of previous studies highlighted the presence of certain phenolic compounds, mainly ferulic acid, gallic acid, and quercetin. *P. sativum* Hoffm. extracts showed no evidence of hepatotoxicity or nephrotoxicity. They demonstrated remarkable anti-inflammatory activity and presented a significant estrogenic effect compared to a negative control group [20]. Parsley administration was observed to partially overcome these issues and resulted in increased superoxide dismutase levels [29]. The antioxidant effect and therapeutic potential of parsley are intensively studied, but not enough lines of applicability have been fully established according to its potential.

It is essential to acknowledge the limitations of this study, including the relatively small sample size and the need for further research to confirm and validate the results. Also, issues related to individual variation in treatment response and possible side effects require careful and extensive investigation.

In this manner, the current results pave the way for further investigations into the use of parsley in various medical contexts. Integrating products obtained from the spontaneous flora into routine treatments can represent an innovative approach to the improvement of muscle and skin health. However, more research is needed to fully understand their potential and the mechanisms involved.

## 4. Materials and Methods

### 4.1. Structure of the Study

The study of paraclinical analysis was conducted in accordance with the research ethics committee of the Faculty of Medicine and Pharmacy, University of Oradea, under protocol no. 12/01.04.2019 within the medical office “Echo Laboratoare”. A retrospective analysis was performed for the period spanning from 2019 to 2022. Before enrollment, each potential participant was provided with an explanation of the study design through a brochure, and an informed consent form was presented. Written informed consent was obtained from each patient before their inclusion in the study, which involved a total of 937 individuals. Inclusion criteria for participation in the study encompassed individuals with psoriasiform atopic dermatitis using parsley ointment and those experiencing muscle cramps with an incidence of at least three times per week. The cream was topically applied for 7 days, and a recommended daily consumption of 20 mL of parsley juice before daily activities was prescribed for a duration of 7 days. Exclusion criteria included psoriasis under allopathic treatment, pathological conditions involving serious liver and kidney diseases, and refusal to participate.

### 4.2. Chemicals, Reagents, and Plant Material

The reagents used in this study were purchased from Elemental (Oradea, Romania), Farmachim 10 SRL (Ploiești, Romania), Sigma-Aldrich (Taufkirchen, Germany), and Fluka (Buchs, Switzerland). The compounds used are of adequate purity, attested by analysis bulletins issued by the manufacturer. Parsley leaves and roots were collected from Bihor County, Romania, in 2019. The leaves were dried and ground into a powder. Lyophilized leaves underwent processing and analysis by GC-MS for the preparation of the ointment, while the root was processed in order to obtain cold-pressed parsley root juice.

### 4.3. Evaluation of Lyophilized Product with Gas Chromatograph (GC-MS)

Sample Preparation: 0.112 g of powder was extracted in 1 mL of ethanol for a day (20–24 h). The extract was analyzed without dilution.

The GC-MS analysis of various organic crude extracts isolated from leaves of *P. crispum* Mill. was conducted using a Thermo GC-MS (Model Trace 1310 ISQ 7000, Waltham, MA, USA) equipped with an HP−5MS capillary column (30 m length × 0.32 mm internal diameter × 0.25 µm film thickness). GC-MS spectroscopic analysis was carried out using an electron ionization system with an ionization energy of 70 eV. Helium served as the carrier gas at 30 cm·s^−1^, with an injection volume of 1 µL. The mass transfer line and injector temperature were set at 220 °C and 290 °C, respectively. The oven temperature was programmed at 45 °C for 1 min, increased to 250 °C at 5 °C min^−1^, and held at 250 °C for 5 min. Diluted samples (1/100, *v*/*v*, in dichloromethane) of 1 µL were injected in the split mode with a split ratio of 120:1. The relative percentage of chemical constituents in the dried extracts of *P. crispum* Mill. was expressed as a percentage based on area normalization.

Qualitative and semi-quantitative analysis was applied to the obtained oil, and the retention indices Kovats were calculated. Retention indices were also applied in characterizing the selectivity of stationary phases, structural analysis, and studies of physico-chemical properties of stationary phases and analytes. The relationships between Kovats indices and thermodynamic properties were used for determining vapor pressures, entropies of adsorption, enthalpies, and the vaporization of different analytes. Following the analysis, detailed results were obtained, as outlined in Table 2. According to the results presented in Table 1, the main substance observed was apiol, constituting 29.22%.

Kovats retention index (***RI***) Equation (1):

***RI*** = 100(***n***) + 100(***m*** − ***n***)(***tr***_***i***_ − ***tr***_***n***_)/(***trm*** − ***tr***_***n***_) (1)

where

- ***RI*** = retention index of “***i***”;
- ***i*** = constituent of essential oil subjected to analysis;
- ***n*** = carbon number of the alkane eluting before “***i***”;
- ***m*** = number of carbons of the alkane eluting after “***i***”;
- ***tr_i_*** = retention time of “***i***”;
- ***tr_n_*** = retention time of the alkane eluting before “***i***”;
- ***tr_m_*** = retention time of the alkane eluting after “***i***”.

### 4.4. Chemical Composition of the Dry Matter

To ensure the antioxidant effect on the skin, an assessment of the heavy metal content in the product was conducted and compared against the standards established by the World Health Organization (WHO) [30].

The samples underwent a drying process in an oven for 16 h at a temperature of 108 °C. Post-drying, they were pulverized and further subjected to calcination at 450 °C for 8 h, resulting in the formation of gray-white ash. They were then solubilized in 6 N HCl and brought to a 100 mL volumetric flask using bidistilled water.

The metal content was determined by atomic absorption spectrometry using a PlasmaQuant MS—ICP-MS Mass Spectrometer (Jena, Germany). The operational conditions were as follows: air-to-acetylene ratio 13.50:2; nebulizer absorption rate 5 L/min (Table 3).

### 4.5. Formulation and Evaluation of Bioadhesive Preparation with Parsley

In formulating a topical preparation, auxiliary substances play a pivotal role in shaping the pharmaceutical product. Excipients significantly impact the physico-chemical properties of the pharmaceutical preparation, influencing its ability to optimally release the active ingredient for the desired therapeutic effect.

Various factors influence the release and absorption of active substances from bioadhesive preparations, including the nature of the ointment base, its capacity for releasing active substances, and the physico-chemical properties of the incorporated substances.

The selection of auxiliary substances and preparation technologies constitute critical measures to ensure robust stability and maximum efficacy at the application site for the bioadhesive preparation. The ointment interacts with the skin, aiding the release of the active ingredient, effortlessly spreading in a thin layer, and adhering effectively to the skin.

For the formulation of a well-tolerated preparation, excipients suitable for the application site, lacking irritating effects and chemically inert, were chosen. In this context, avocado butter and olive wax were selected as excipients.

**Figure 5:**
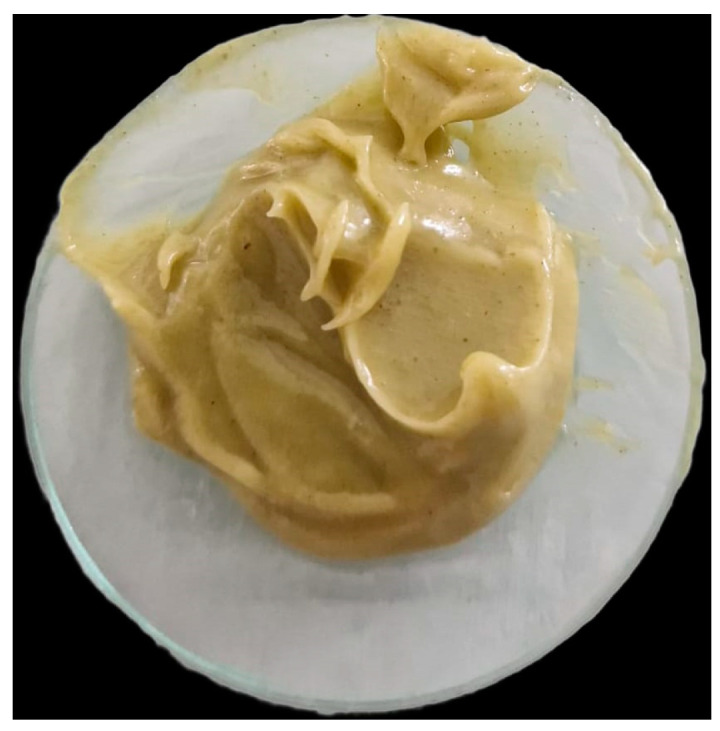
The bioadhesive products with urea (PBU).

Considering the physico-chemical characteristics of the active ingredient, successful inclusion in a fat base was achieved, as depicted in Figure 5.

#### Preparation of Bioadhesive Products

The preparations were obtained in two stages. Initially, the ointment base was prepared by melting beeswax and avocado butter in a saucepan. The parsley powder was incorporated into the melted mixture by suspension. Homogenization continued until the preparation cooled, resulting in the PB (bioadhesive product *sine* urea) formula. Subsequently, the PBU formula was obtained by suspending the parsley powder in the ointment base, followed by the incorporation of the urea solution through emulsification.

Avocado butter, a solid and soft butter with natural green notes, enhances hydration, softens the skin, and provides protection against dryness. Being rich in vitamins, phytosterols, and unsaturated fatty acids, it improves the skin’s barrier function and leaves a comforting layer on the skin.

Olive wax, a natural water-in-oil emulsifier, presented in cream-yellow flakes soluble in oil, offers multifunctional benefits as a powder dispersant and is biodegradable.

### 4.6. Quality Control of Bioadhesive Preparations

In order to assess the effectiveness, quality, and stability of the bioadhesive preparations, various determinations of the characteristics of ointments were performed over a period of time. These included organoleptic examination, determination of homogeneity (using a 4.5× magnifying glass), pH determination (potentiometric method), and spreadability assessment (extensiometric method—Ojeda Arbussa method). These determinations were conducted on freshly prepared samples and after 30 and 60 days. No change in the properties of the ointment was observed.

The protective capacity of the skin was monitored through topical applications, comparing it to a cream without parsley. The pH of the formulation with parsley was measured at 5.48, while the one without parsley was measured at 6.05.

**Figure 6:**
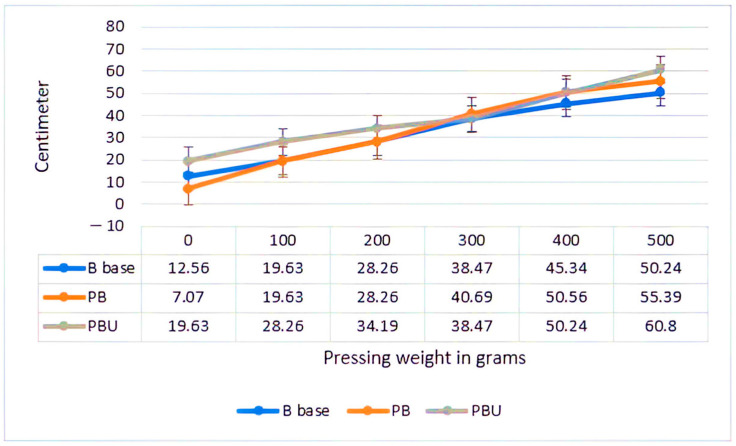
The spreadability graph illustrates the performance of both the ointment base and the two parsley formulas.

A figure of 3% urea was incorporated to enhance spreadability and adjust the pH. The spreadability graph illustrates the performance with both the ointment base and the two parsley formulas (Figure 6).

### 4.7. Immunological Tests

The microarray-based enzyme immunoassay (M-EIA) utilized for immunological testing detects IgG antibodies against food allergens. Extracted and purified food extracts are applied to a microarray. The RIDA^®^CHIP FoodGuide consists of multiple food allergens in each cavity, including two standard rows for quantification and positive and negative controls. Patient samples (capillary blood eluate) were pipetted into wells and incubated, allowing specific IgG antibodies to bind to the corresponding adsorbed food antigens. After washing to remove unbound material, an anti-human IgG antibody conjugated with horseradish peroxidase was added. This conjugate bound itself to human IgG antibodies in the patient sample during reincubation. Unbound conjugate was washed away, and once the substrate was added, an insoluble blue product was formed through the oxidized horseradish peroxidase [31]. The amount of blue precipitate was proportional to the antigen-specific antibodies in the serum, detectable photographically. RIDASOFT^®^ Win.Net Food&Feet A8592 was used to quantify the resulting data, generating comprehensive reports. Calibration was carried out using standard solutions with concentrations between 0.3 and 3 μg/L, prepared from an ICP multi-element standard solution of 1000 mg/L (Merck KGaA, Darmstadt, Germany).

### 4.8. Muscle Cramp Verification Test

The efficacy of cold-pressed parsley juice was verified by numerically assessing the frequency of muscle cramps initially and after 7 days.

### 4.9. Dermatological Evaluation

Clinical evaluation of dermatitis severity (erythema, redness, dryness) was conducted at baseline, during and at the end of the treatment using scores for erythema, induration, and desquamation on a scale of 0 to 4.

### 4.10. Statistical Analysis

The study’s exit rate was 0.00%, and Chi-square tests were employed to identify lots with statistical significance (Asymp. Sig. < 0.05). The statistical analysis was accomplished using the software SPSS Statistics 22. Mean values, frequency intervals, standard deviations, and tests of statistical significance were calculated using the Student’s *t*-test. Distribution of lots was assessed to be similar to the normal distribution, involving assumptions with numerical data. The Bravais–Pearson correlation coefficient determined an independent indicator of the units of measurement for two variables. A significance level of *p* < 0.05 was assigned to ANOVA, and *p* < 0.01 indicated high-level statistical significance. Bonferroni post hoc analysis was used for subgroup analysis of differences between groups.

## 5. Conclusions

Parsley presented high tolerability in patients, with 96.26% reporting no significant immunological reactions. This result underscores the potential inclusion of parsley in individual diets without major adverse effects on the immune response.

The consumption of cold-pressed parsley juice demonstrated a significant reduction in the incidence of muscle cramps, with a 15.02% decrease in cases. This observation suggests that parsley juice may offer potential benefits in the management or prevention of muscle cramps, thus offering potential strategies for addressing these symptoms.

The use of the ointment exhibited a significant reduction in aspects such as redness, thickness, and scaling in individuals with dermatological problems. These results imply that the ointment may serve as an effective alternative in the treatment of various forms of dermatitis. However, to confirm and validate these findings, further studies are required to thoroughly investigate the long-term impact and efficacy of the ointment in the treatment of dermatological conditions.

## Figures and Tables

**Table 1:** Statistical description of the evolution of initial and final dermatological problems (after 7 days of application).

Parameters		Groups				Total	*p*
		Control Group		Study Group			
		Count	%	Count	%		
Redness initial	0%	0	0.0	0	0.0	36	0.002 **
	<10%	3	9.4	5	15.6		
	10–29%	8	25.0	7	21.9		
	30–49%	5	15.6	4	12.5		
*p*		0.477					
Redness final	0%	0	0.0	0	0.0		
	<10%	4	12.5	10	31.2		
	10–29%	7	21.9	5	15.6		
	30–49%	5	15.6	1	3.1		
*p*		0.020 *					
Thickness initial	0%	0	0.0	0	0.0		0.001 **
	<10%	6	18.8	6	18.8		
	10–29%	7	21.9	8	25.0		
	30–49%	3	9.4	2	6.2		
*p*		0.837					
Thickness final	0%	0	0.0	1	3.1		
	<10%	6	18.8	12	37.5		
	10–29%	8	25.0	3	9.4		
	30–49%	2	6.2	0	0.0		
*p*		0.008 **					
Scaling initial	0%	0	0.0	0	0.0		0.001 **
	<10%	5	15.6	8	25.0		
	10–29%	7	21.9	5	15.6		
	30–49%	4	12.5	3	9.4		
*p*		0.343					
Scaling final	0%	1	3.1	4	12.5		
	<10%	5	15.6	10	31.2		
	10–29%	6	18.8	2	6.2		
	30–49%	4	12.5	0	0.0		
*p*		0.003 **					

*p* = statistically significance, * correlation is significant at the 0.05 level, ** correlation is significant at the 0.01 level.

**Table 2:** Gas chromatograph report (g × 100 g^−1^ DW) for the parsley extract in percentage composition.

Product	Substance	Retention Time (min)	Peak Number	Kovats Index
Green parsley powder	Acetic acid	2.88	9	Reference
	Thiourea	3.40	10	18.05
	Triethylfluorosilane	4.06	11	40.97
	1,2,3,4-diepoxybutane	4.35	12	51.04
	Hydrazoic acid	4.55	13	57.98
	N-Methoxyformamide	5.16	14	79.16
	Glyceraldehyde	5.46	15	89.58
	Pyruvic acid, methyl ester	6.59	16	129.81
	2-Methylcyclopentanol	6.72	17	133.33
	Beta-Myrcene	9.15	19	217.70
	1-Isopropyl-4-methylenebicyclo(3,1,0)-hexane	10.10	20	250.69
	Maltol	11.20	21	288.88
	p-Cymenene	11.59	22	302.43
	p-Mentha-1,3,8-triene	12.16	23	322.22
	1-Vinyl-cyclohexanol	12.39	24	330.20
	Glyceraldehyde	12.73	25	342.01
	3,5-Dihydroxy-6-methyl-2,3-dihydro-4H-pyran-4-one	12.88	26	347.22
	3-Methylacetophenone	13.93	27	383.68
	5-Hydroxymethylfurfural	14.87	28	416.31
	1-Acetate-1,2,3-propanetriol	15.18	29	427.08
	8a-Chlorooctahydro-1(H)-naphthalenone	18.81	31	553.12
	m-Cymen-8-ol	18.92	32	556.94
	3-Isopropenyl-2,5-dimethyl-3,4-hexadien-2-ol	20.98	34	628.47
	5-allyl-1-methoxy-2,3-(methylenedioxy)-benzene	21.42	35	643.75
	Sesquisabinenes isomer	21.47	36	645.48
	5,6,7,7a-tetrahydro-4,4,7A-trimethyl-2(4H)-benzofuranone	21.67	37	652.43
	3-Deoxy-d-mannoic-lactones	22.46	-	679.86
	1,2,3,5-Cyclohexanetetrol	22.88	38	694.44
	Heptose	23.04	39	700.00
	Apiol	24.51	40	751.04
	6-Hydroxy-4,4,7a-trimethyl-5,6,7,7a-tetrahydrobenzofuran-2(4H)-one	26.06	41	804.86
	Ficusin	27.12	42	841.66
	Neophytadians	27.21	43	844.79
	3,7,11,15-Tetramethyl-2-hexanedecen-1-ol	27.63	44	859.37
	Hexanoic acid-2-phenylethyl ester	27.94	45	870.13
	Palmitic acid	29.20	47	913.88
	Methoxsalen	30.59	49	962.15
	Bergapten	30.92	50	973.61
	Pythol	31.62	53	998.26
	Linoleic acid	31.88	54	1006.94
	Linolenic acid	31.99	55	1010.76
	1-Methyl-8-(1-methylethyl)-tricyclo[4,4,0,0(2,7)]dec-3-ene-3-methanol	32.10	56	1014.58
	1-Methyl-3[(1-methylethyldiene)-cyclopropyl]-benzene	32.19	57	1017.70
	Stearic acid	32.30	58	1021.52
	Octanoic acid 2-dimethylaminoethyl ester	34.10	59	1084.02
	8-13-epoxy-1,15,16-trinor-8-xi-labdan-6beta-ol	34.35	60	1092.70
	3-Cyclopentylpropionic acid 2-dimethylaminoethyl ester	36.43	61	1164.93
	Oxypeucedarin	36.83	63	1178.81
	2-Monopalmitin	37.13	64	1189.23

**Table 3:** Operating conditions for the Varian Spectra 240 FS spectrophotometer.

Metal	λ (nm)	Lamp Current (mA)	Slit Width	WHO Standard
Ni	232	4	0.2	Not specified
Cr	357.9	7	0.2	Not specified
Cu	324.8	4	0.5	Not specified
Cd	228.8	4	0.5	0.005 ppm
Mn	279.5	5	0.2	0.1 ppm
Zn	213	5	1	Not specified
Fe	248.3	5	0.2	0.3 ppm
Pb	283.3	10	1.2	0.05 ppm

## Data Availability

The original contributions presented in the study are included in the article, further inquiries can be directed to the corresponding author.
